# Impact of accelerometer epoch summary measure on associations between physical activity and all-cause mortality in Whitehall II and UK Biobank

**DOI:** 10.1038/s41598-025-30237-5

**Published:** 2025-12-06

**Authors:** Henrik R. Eckmann, Cameron Razieh, Ian Meneghel Danilevicz, Sam Vidil, Sebastien Chastin, Lauren B. Sherar, Bjørge H. Hansen, Paddy C. Dempsey, Séverine Sabia, Alex V. Rowlands

**Affiliations:** 1 Diabetes Research Centre, Leicester General Hospital, University of Leicester, Leicester, LE5 4PW UK; 2 National Institute for Health Research (NIHR) Leicester Biomedical Research Centre (BRC), University Hospitals of Leicester NHS Trust and The University of Leicester, Leicester, LE5 4PW UK; 3 Leicester Real World Evidence Unit, Diabetes Research Centre, Leicester General Hospital, University of Leicester, Leicester, LE5 4PW UK; 4 Office for National Statistics, Newport, NP10 8XG UK; 5 Epidemiology of Ageing and Neurodegenerative Diseases, Universite Paris Cité, INSERM, U1153, CRESS, 10 Avenue de Verdun, 75010 Paris, France; 6 School of Health and Life Sciences, Glasgow Caledonian University, Glasgow, G4 0BA UK; 7 Department of Movement and Sports Sciences, Ghent University, Watersportlaan 2, 9000 Ghent, Belgium; 8 School of Sport, Health and Exercise Sciences, Loughborough University, Loughborough, LE11 3TU UK; 9 Department of Sport Science and Physical Education, University of Agder, P.O. Box 422, 4604 Kristiansand, Norway; 10 Institute for Physical Activity and Nutrition (IPAN), School of Exercise and Nutrition Sciences, Deakin University, 221 Burwood Highway, Burwood, Victoria 3125 Australia; 11 Baker Heart and Diabetes Institute, PO Box 6492, Melbourne Victoria, 3004 Australia; 12 MRC Epidemiology Unit, Institute of Metabolic Science, University of Cambridge, Cambridge Biomedical Campus, Cambridge, CB2 0SL UK; 13 UCL Brain Sciences, Division of Psychiatry, University College London, Russell Square House, 10-12 Russell Square, London, WC1B 5EH UK; 14 Alliance for Research in Exercise, Nutrition and Activity (ARENA), UniSA Allied Health and Human Performance, University of South Australia, Adelaide, 5001 Australia

**Keywords:** Average acceleration, Intensity gradient, Epoch summary measure, Physical activity, Mortality, Accelerometry, Biomarkers, Cardiology, Diseases, Health care, Medical research, Risk factors

## Abstract

Accelerometer data are commonly reduced into epoch summary measures (ESMs) for analysis, e.g. ENMO (Euclidean Norm Minus One), MAD (Mean Amplitude Deviation), MIMS (Monitor Independent Movement Summary) or Counts. We compared associations with all-cause mortality of the volume and intensity of physical activity when derived from those four measures in the Whitehall II and UK Biobank cohorts. Volume (Average Acceleration, AvAcc) and intensity (Intensity Gradient, IG) were derived from each ESM. Associations with mortality were estimated using Cox models. 3733 (25.1% female, median age 68.3 years) and 89,848 (56.4% female, 63.5 years) participants were included from Whitehall II and UK Biobank, respectively. Median (IQR) follow-up was 11.0 (10.7, 11.3) and 8.0 (7.5, 8.5) years, with 563 (15.1%) and 3656 (4.1%) deaths. Associations with mortality were largely consistent between ESMs with the lowest mortality risk for those high (above the median) in both AvAcc and IG (Whitehall: HR = 0.59–0.68; Biobank: 0.55–0.61, reference: low/low), and IG associated with lower mortality risk, irrespective of AvAcc. AvAcc was associated with lower mortality irrespective of IG in Biobank only. In conclusion, associations of AvAcc and IG with mortality are broadly consistent across common ESMs, supporting comparability of activity-health findings across studies using different ESMs.

**Supplementary Information:**

The online version contains supplementary material available at 10.1038/s41598-025-30237-5.

## Introduction

Since 2010, accelerometer-based assessment of physical activity (PA) has become increasingly accessible, even for large-scale studies^1^, enabling a more detailed understanding of the relationships between PA and health e.g. life expectancy^2^, incident cardiovascular disease (CVD)^3^, and all-cause mortality^4,5^.

Among PA variables, the Average Acceleration (AvAcc) of accelerometer-assessed PA across the day is a proxy for PA volume^6^, and the Intensity Gradient (IG) describes how PA is accumulated over the day—i.e. mainly at lower or higher intensities^7^. This combination of PA volume and intensity explains more variance in cardiometabolic risk^8^, waist-to-height-ratio and metabolic syndrome risk^9^, incident cardiovascular disease^3^, and life expectancy^2^ than either alone.

Accelerometers store high resolution accelerations. These accelerations result not only from movement, but also from gravity and sensor noise. To convert the raw data into meaningful PA variables (e.g., AvAcc and IG), primarily reflecting accelerations due to movement, the acceleration data are processed and summarised into an epoch summary measure (referred to herein simply as “measure”)^10^. Pre-2010, this processing was carried out on-board the accelerometer and only manufacturer-specific “counts” were available to researchers^11^. Post-2010, accelerometer manufacturers made the raw accelerations available to researchers who could select from open-source measures, such as the Euclidean Norm Minus One (ENMO)^10^ or the Mean Amplitude Deviation (MAD)^12^. The standard variant and a filtered variant of ENMO were used in the German National Cohort Study (NAKO) and UK Biobank^13^, respectively, with MAD used as a secondary measure in NAKO^14^, the Monitor Independent Movement Summary (MIMS)^15^, has been used in the National Health and Nutrition Examination Survey (NHANES)^16^, and the ActiGraph Counts algorithm (referred to herein as “Counts”) has been published^17^.

As diverse measures proliferate, it is critical to know whether PA–health associations hold regardless of measure choice. Recently, Willems et al. showed AvAcc and IG, and their correlation to cardiometabolic health markers, can vary between measures^18^. This likely relates to key differences and considerations relating to the intrinsic properties of the measures^19^, with Karas et al. reporting non-linear relationships between commonly used measures^20^. Further, the IG was originally developed for use with ENMO^7^. Similarly to Willems et al.,^21–23^ found limited agreement between IGs derived from different measures. However, some improvement was evident following measure-specific adjustments to the IG algorithm to adapt it for use with MAD, MIMS, and Counts^23^. It is not known whether measure-specific adjustments to IG yield comparable AvAcc-IG-mortality associations across ENMO, MAD, MIMS, and Counts.

This study aimed to compare combined associations of AvAcc and IG with mortality computed from four measures (ENMO, MAD, MIMS, and Counts) in two large cohorts—Whitehall II^24,25^ and UK Biobank^13,26^—where combined associations with mortality of PA volume and intensity generated from the ENMO measure have previously been demonstrated^2,27^. This study is part of the Learning Network for Advanced Behavioural Data Analysis (LABDA)^28^.

## Materials and methods

### Data sources and study populations

Established in 1985, the Whitehall II study aims to investigate health inequalities in a cohort of 10,314 British civil servants (6900 men, 3414 women)^29^. Recurrent data collections have been conducted in the study roughly every five years, with accelerometry added in a subpopulation of 4282 participants in 2012–2013. Written, informed consent from participants was obtained at each contact. Research ethics approvals were renewed at each wave; the most recent approval was granted by the National Health Service (NHS) London-Harrow Research Ethics Committee (reference number 85/0938). Participants wore a GENEActiv (Activinsights Ltd, Cambridgeshire, UK) accelerometer continuously for nine days, sampling at 85.7 Hz, $\pm$ 8 *g* (gravitational unit (1 *g* = 9.81 m/s^2^)), on their non-dominant wrist^24^.

UK Biobank consists of 500,000 British participants aged 40–69 recruited in 2006–2010^26^. UK Biobank has full ethical approval from the NHS National Research Ethics Service (16/NW/0274) and all participants gave written informed consent prior to data collection. Between 2013 and 2016 accelerometry was collected in a subpopulation of 106,053 participants continuously for seven days, employing the Axivity AX3 device (Axivity Ltd, Newcastle, UK), sampling at 100 Hz, $\pm$ 8 *g*, worn on the dominant wrist^13^.

Besides accelerometry, both datasets contain clinical, questionnaire, and health registry linked data, including mortality data^26,30^.

All procedures and methods conform to the ethical guidelines defined by the World Medical Association’s Declaration of Helsinki and its subsequent revisions.

### Accelerometer processing

Accelerometer data were initially processed using the R package GGIR^31^ part 1 with default settings, including autocalibration^32^, non-wear and clipping detection^31^ to generate epoch-level data for ENMO, MAD, and Counts in 5 s epochs. Five second epochs were used in the original implementation of the IG^7^, has commonly been used in comparable analyses^2–5^, and has been shown to be more appropriate for use with the IG, compared to longer epochs^33^. Code recreating relevant steps in GGIR part 1, or calling specific functions within GGIR part 1 where possible, was then applied to generate MIMS using the MIMSUnit R package^15^ and integrate the output with the GGIR part 1 output to feed into GGIR part 2. In GGIR part 2, invalid data (non-wear or clipping) was imputed using the average of the same point in time across the remaining measurement days per measure. Participants failing calibration (> 0.01 *g* calibration error^7^), with fewer than 3 valid days (> 16 h/day) of wear, or where wear-data were not present for each 15-min period of the 24-h cycle, were excluded^7^.

#### Epoch summary measures

ENMO is the epoch mean of the vector magnitude of the triaxial acceleration signal, minus 1 *g* to account for the influence of gravity, with negative values rounded to zero. It is thus a measure of the mean magnitude of acceleration within the epoch^10^. MAD is likewise based on the vector magnitude but measures the mean absolute deviation from the epoch mean, as opposed to the epoch mean directly^12^. Counts and MIMS are both derived from more complex algorithms consisting mainly of multiple resampling and filtering steps, meant to account for differences in sampling rate, and/or gravity and signal noise^15,17^. More details regarding each measure are included in Supplementary Section S1.

#### Physical activity exposures

AvAcc and IG were generated for each measure per valid 24-h day, using the adjusted bins proposed previously for IG^23^. Note, Eckmann et al. tested two types of adjusted bins, “modelled” and “naïve”, with the “modelled” approach providing better agreement between measures. Therefore, in our analysis the “modelled” bins were used. AvAcc and IG per participant were each then calculated as the mean of the valid days available for each participant. Participants with AvAcc values, based on ENMO, above 100 m*g* were excluded from all analyses, including those based on MAD, MIMS, and Counts, as these were considered to be unrealistic, and therefore indicative of errors in the raw acceleration file, consistent with previous UK Biobank analyses^34^. Additionally, participants above and below the 99.5th and 0.5th percentile, respectively, for either of AvAcc or IG based on ENMO were also excluded from all analyses to limit the influence of outliers. Exclusions were applied for all analyses based on ENMO only to keep the dataset consistent for comparison between measures, and because thresholds for unrealistic values are not available for all of the epoch summary measures considered.

### Covariate measurement

Covariates for this analysis included socio-demographic and lifestyle related characteristics of age, sex, ethnicity (white/non-white), education level (low, middle, high), current employment (yes/no), self-reported sleep duration (< 7, 7–8, > 8 h), smoking status (current, former, never), and alcohol consumption (frequency, Whitehall II: never, < 1/month, 2–4/month, 2–3/week, 4+/week; UK Biobank: never, < 1/month, 1–3/month, 1–2/week, 3–4/week, 4+/week). Health-related covariates included body mass index (BMI) (underweight, normal, overweight, obese), number of prescribed medications, prevalent CVD, cancer, and number of other prevalent chronic diseases. Season of accelerometer wear was derived from the start time of the accelerometer recording using two orthogonal sine functions as in Dempsey et al.^3^

For Whitehall II, covariates were extracted from a clinical screening and questionnaire at the time of accelerometer data collection and imputed with answers from previous screenings/questionnaires where missing. Disease and risk factor covariates were updated if Hospital Episode Statistics (HES) data included a diagnosis before accelerometer screening date. For UK Biobank, all participants completed a questionnaire and assessment at recruitment into the main study, with some participants completing two further follow-up questionnaires. Where available, covariate data from the most recent questionnaire before accelerometer assessment were used, and baseline data were used otherwise. Strain et al. showed mostly minor differences between covariates measured at baseline versus later questionnaires^4^. Sex was only obtained at recruitment baseline, and ethnicity was assumed to be constant. Covariates were kept as consistent as possible between both cohorts, but some differences were unavoidable, details are included in Supplementary Table S1. Participants with missing data were excluded.

### Outcome

The outcome of interest was all-cause mortality identified from the UK Office for National Statistics Mortality Register. Participants were censored at time of death or censoring (October 2023 Whitehall II; November 2022 UK Biobank).

### Statistical analyses

Descriptive statistics are reported as median and interquartile range (IQR) for continuous variables and number and percentage (%) for categorical variables.

Relative agreement between measures for AvAcc and for IG was assessed using Pearson’s *r*. The Intraclass Correlation Coefficient^35^ (ICC, two-way mixed effects model, consistency, single rater) was also estimated for IG only. The ICC is sensitive to differences in both mean and scale between measures^35^ and would therefore not be a good measure of agreement for AvAcc, as AvAcc inherits scale and unit from a given measure.

Both AvAcc and IG, for each measure, were modelled as natural splines with three internal knots (boundary knots at the 5th and 95th percentile, internal knots at the 25th, 50th and 75th percentile of values within the boundary knots) in mutually adjusted Cox proportional hazards model, and HRs were plotted as a function of each. Linear terms and splines with one through seven internal knots were tested. Linear terms and splines with fewer than three internal knots caused proportionality and/or linearity to fail across several models. More knots uniformly resulted in higher model Bayesian Information Criterion values (Supplementary Figure S1). To be able to compare between measures, both AvAcc and IG were standardised, for each measure, before modelling by subtracting the mean and dividing by the standard deviation such that the value represents the number of standard deviations from the mean.

To quantify the combined impact of low versus high AvAcc and IG, for each measure, including interactions between the two, a new categorical variable was created categorising participants into one of four categories based on whether their AvAcc and IG were above or below the median, i.e. low AvAcc and low IG (“low/low”), low AvAcc and high IG, high AvAcc and low IG, and high AvAcc and high IG. This categorical variable was then used as the main exposure in a second Cox model with low/low as reference and hazard ratios (HR) were estimated.

Age was used as the underlying timescale. The main analyses were adjusted for only hypothesised confounders, with an additional sensitivity analysis undertaken to include potential mediators. All socio-demographic and lifestyle covariates were considered confounders, along with season of accelerometer wear. All health-related covariates were considered possible mediators or confounders (Supplementary Figure S2). Proportional hazards and linearity assumptions were checked using Schoenfeld residuals and Martingale residuals respectively; covariates that failed to meet the proportionality assumption were used to stratify baseline hazards. A further sensitivity analysis including only participants aged ≥ 60 at time of accelerometer measurement in UK Biobank was carried out to match the age range to the Whitehall II cohort.

Analyses were replicated in both Whitehall II and UK Biobank and completed using R version 4.3.1. UK Biobank data were processed using ALICE High Performance Computing at the University of Leicester. Results are reported with 95% confidence intervals (CI). All relevant code is available on GitHub at github.com/henrikeckmann.

## Results

### Sample characteristics

Of 4,210 available accelerometer files for Whitehall II, 3,733 participants were left after exclusions, including 939 (25.1%) women. More details regarding exclusions are included in Supplementary Figure S3. Median age (median (IQR)) was 68.3 (64.7, 73.8), and 3,497 (93.7%) participants were white. Over a median follow-up of 11.0 (10.8, 11.3) years, 563 (15.1%) deaths occurred, 453 (16.2%) in men, and 110 (11.7%) in women.

For UK Biobank, of an initial 115,409, 89,848 participants were left after exclusions, including 50,710 (56.4%) women. Median age was 63.5 (56.3, 68.6) and 87,118 (97.0%) participants were white. Over a median follow-up of 8.0 (7.5, 8.5) years, 3656 (4.1%) deaths occurred, 2188 (5.6%) in men, and 1468 (2.9%) in women. Descriptive statistics for covariates and PA measures are included in Tables 1 and 2.

**Table 1:** Sample characteristics.

	Whitehall II	UK Biobank
n (%)	3733 (100%)	89,848 (100%)
Age, years	68.3 (64.7, 73.8)	63.5 (56.3, 68.6)
White ethnicity	3497 (93.7%)	87,118 (97.0%)
Currently in employment	720 (19.3%)	55,642 (61.9%)
Smoking		
Never	1817 (48.7%)	51,363 (57.2%)
Former	1801 (48.2%)	32,353 (36.0%)
Current	115 (3.1%)	6132 (6.8%)
Alcohol frequency		
Never	115 (3.1%)	5000 (5.6%)
< 1/month	465 (12.5%)	8431 (9.4%)
1–3/month	N/A	9767 (10.9%)
2–4/month	658 (17.6%)	N/A
1–2/week	N/A	22,572 (25.1%)
2–3/week	1,003 (26.9%)	N/A
3–4/week	N/A	23,459 (26.1%)
4+/week	1492 (40.0%)	20,619 (22.9%)
Avg. sleep duration		
< 7 h	1416 (37.9%)	19,669 (21.9%)
7–8 h	2218 (59.4%)	64,544 (71.8%)
> 9 h	99 (2.7%)	5635 (6.3%)
No. of prescribed medications*	3 (1, 5)	0 (0, 1)
BMI		
< 18.5 kg/m^2^	45 (1.2%)	487 (0.5%)
18.5–25 kg/m^2^	1380 (37.0%)	34,936 (38.9%)
25–30 kg/m^2^	1635 (43.8%)	37,147 (41.3%)
> 30 kg/m^2^	673 (18.0%)	17,278 (19.2%)
Prevalent disease		
CVD*	605 (16.2%)	6287 (7.0%)
Cancer*	389 (10.4%)	12,610 (14.0%)
Long-standing illness or disease	N/A	24,777 (27.6%)
No. of other diseases*	1 (0, 1)	0 (0, 0)

Median (Q1, Q3) or n (%).

*Slight differences in definition between cohorts. See Supplementary Table S1 for details.

*BMI* Body Mass Index, *CVD* Cardiovascular Disease0.

**Table 2:** Mortality and physical activity characteristics.

	Whitehall II	UK biobank
Median follow-up time, years	11.0 (10.7, 11.3)	8.0 (7.5, 8.5)
Follow-up time, total person-years	39,154	713,771
Deaths by cause		
Total	563 (15.1%)	3656 (4.1%)
CVD deaths	100 (17.8%)	743 (20.3%)
Cancer deaths	247 (43.9%)	2022 (55.3%)
Respiratory deaths	45 (8.0%)	302 (8.3%)
Other deaths	171 (30.4%)	589 (16.1%)
Age at death, years	80.7 (76.1, 85.1)	73.7 (69.1, 77.4)
Accelerometry		
Calibration error, g	0.003 (0.002, 0.003)	0.004 (0.003, 0.004)
Valid wear days	8 (8, 8)	6 (6, 6)
AvAcc		
ENMO, mg	22.9 (19.2, 27.3)	28.5 (23.8, 34.1)
MAD, mg	39.6 (33.6, 46.1)	43.1 (36.6, 50.3)
MIMS	0.67 (0.57, 0.77)	0.80 (0.69, 0.92)
Counts	82.1 (63.8, 103.6)	158.6 (134.1, 185.2)
IG		
ENMO	− 2.704 (− 2.821, − 2.575)	− 2.560 (− 2.668, − 2.443)
MAD	− 3.083 (− 3.194, − 2.973)	− 2.999 (− 3.090, − 2.897)
MIMS	− 4.097 (− 4.238, − 3.942)	− 3.853 (− 3.997, − 3.694)
Counts	− 3.362 (− 3.501, − 3.219)	− 3.485 (− 3.572, − 3.386)

Median (Q1, Q3) or n (%).

*Slight differences in definition between cohorts. See Supplementary Table S1 for details.

AvAcc: Average Acceleration, IG: Intensity Gradient, ENMO: Euclidean Norm Minus One, MAD: Mean Amplitude Deviation, MIMS: Monitor Independent Movement Summary.

### Relative agreement of AvAcc and IG across ENMO, MAD, MIMS, and counts

#### Whitehall II

For AvAcc, correlations ranged from 0.79 (CI: 0.78, 0.80) between MIMS and Counts, to 0.98 (0.98, 0.98) between ENMO and MAD (Table 3). The strongest correlation was between ENMO and MAD, while the weakest were when Counts was one of the measures. For IG, patterns for Pearson’s *r* and ICC were similar. ICCs ranged from 0.59 (0.57, 0.61), between MAD and Counts, to 0.88 (0.88, 0.89), between ENMO and MAD, indicating moderate to good agreement^35^. Consistent with AvAcc, the strongest correlation was between ENMO and MAD, while the weakest were when Counts was one of the measures.

**Table 3:** Physical activity variable correlations.

	Whitehall II			UK Biobank		
	Counts	MIMS	MAD	Counts	MIMS	MAD
AvAcc, *r*						
MIMS	0.79 (0.78, 0.80)			0.79 (0.79, 0.80)		
MAD	0.81 (0.79, 0.82)	0.89 (0.89, 0.90)		0.87 (0.87, 0.87)	0.72 (0.72, 0.73)	
ENMO	0.80 (0.79, 0.81)	0.87 (0.86, 0.88)	0.98 (0.98, 0.98)	0.84 (0.84, 0.84)	0.69 (0.69, 0.70)	0.97 (0.97, 0.97)
IG, ICC						
MIMS	0.69 (0.67, 0.71)			0.69 (0.69, 0.70)		
MAD	0.59 (0.57, 0.61)	0.69 (0.68, 0.71)		0.56 (0.55, 0.56)	0.72 (0.72, 0.73)	
ENMO	0.69 (0.67, 0.71)	0.80 (0.79, 0.81)	0.88 (0.88, 0.89)	0.60 (0.59, 0.60)	0.80 (0.80, 0.80)	0.87 (0.87, 0.87)
IG, *r*						
MIMS	0.69 (0.67, 0.71)			0.76 (0.76, 0.76)		
MAD	0.61 (0.59, 0.63)	0.74 (0.72, 0.75)		0.56 (0.55, 0.56)	0.79 (0.79, 0.80)	
ENMO	0.70 (0.69, 0.72)	0.83 (0.82, 0.84)	0.89 (0.88, 0.89)	0.60 (0.59, 0.60)	0.84 (0.84, 0.85)	0.87 (0.87, 0.87)

Estimate (95% confidence interval).

AvAcc: Average Acceleration, IG: Intensity Gradient, ENMO: Euclidean Norm Minus One, MAD: Mean Amplitude Deviation, MIMS: Monitor Independent Movement Summary, ICC: Intra-class coefficient, *r*: Pearson correlation coefficient.

#### UK Biobank

For AvAcc, correlations were generally similar to Whitehall II, except for lower correlations for MIMS with ENMO or MAD. Correlations ranged from 0.69 (0.69, 0.70) between MIMS and ENMO, to 0.97 (0.97, 0.97) between ENMO and MAD. As with Whitehall II, the strongest correlation was for ENMO and MAD, however, here the weakest correlations were where MIMS was one of the measures, while in Whitehall II Counts showed the weakest correlations. For IG, patterns for Pearson’s r and ICC were similar. ICCs ranged from 0.60 (0.59, 0.60), between ENMO and Counts, to 0.87 (0.87, 0.87), between ENMO and MAD, indicating moderate to good agreement^35^. Consistent with AvAcc and Whitehall II, ENMO and MAD had the strongest correlation. Consistent with Whitehall II, but not AvAcc in UK Biobank, Counts showed the weakest correlations with the other measures.

### Associations of PA volume and intensity with mortality

#### Whitehall II

**Fig. 1:**
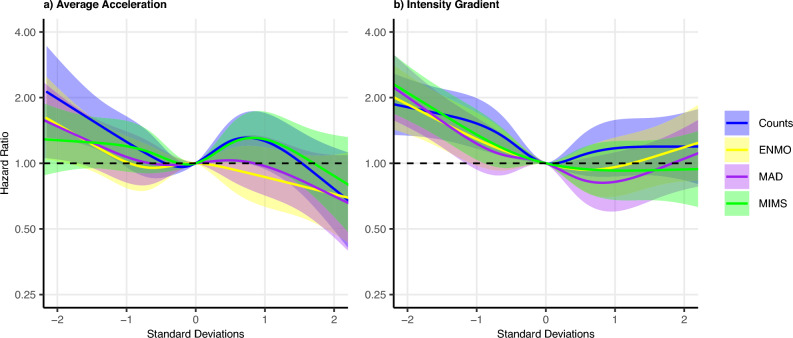
Hazard ratios for mortality according to (**a**) AvAcc and (**b**) IG, from ENMO, MAD, MIMS, and Counts, Whitehall II. X-axis: Number of standard deviations above or below the mean, y-axis: Hazard ratio for all-cause mortality, shaded areas correspond to 95% confidence intervals. AvAcc: Average acceleration, IG: Intensity Gradient, ENMO: Euclidean Norm Minus One, MAD: Mean Amplitude Deviation, MIMS: Monitor Independent Movement Summary. Models adjusted for sex, season, ethnicity, employment status, smoking, alcohol consumption frequency, and sleep duration. See Supplementary Table S1 for details.

Mortality risk was lower for higher values of AvAcc from ENMO, with a plateau between approximately − 1 to 0 standard deviations (SDs) (Fig. 1a). The pattern from the alternate measures was broadly similar with all CIs overlapping throughout the range, with the pattern for AvAcc from MAD most similar. In contrast to ENMO and MAD, for both Counts and MIMS, the HR tended to be higher between 0 and 1 SDs than either side of the interval.

The risk of mortality likewise was lower for higher values of IG from ENMO, with a plateau between 0 and 1 SD, and slightly higher HRs for higher values of IG from ENMO above 1 SD although all CI in this region overlap with 1 (Fig. 1b). The remaining IGs all showed similar patterns with CIs overlapping throughout the range, except for the HR being higher for higher values of IG from Counts between 0 and 1 SD, and no change above 1 SD.

In comparison to AvAcc, observed HRs for IG tended to be higher in the region below the mean, except for Counts where the pattern of the association was similar to AvAcc.

#### UK Biobank

**Fig. 2:**
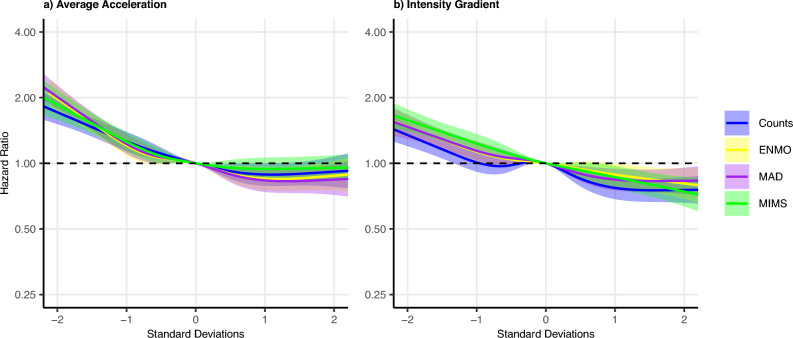
Hazard ratios for mortality according to (**a**) AvAcc and (**b**) IG from ENMO, MAD, MIMS, and Counts, UK Biobank. X-axis: Number of standard deviations above or below the mean, y-axis: Hazard ratio for all-cause mortality, shaded areas correspond to 95% confidence intervals. AvAcc: Average acceleration, IG: Intensity Gradient, ENMO: Euclidean Norm Minus One, MAD: Mean Amplitude Deviation, MIMS: Monitor Independent Movement Summary. Models adjusted for sex, season, ethnicity, employment status, smoking, alcohol consumption frequency, and sleep duration. See Supplementary Table S1 for details.

Mortality risk was lower for higher values of AvAcc from ENMO, with a slight plateau starting just below the mean (Fig. 2a). HRs for AvAcc from MAD, MIMS, and Counts all followed a similar pattern, with CIs overlapping throughout the range. There was greater consistency between measures than observed for Whitehall II.

The HR likewise was lower for higher values of IG from ENMO, again with a slight plateau starting around the mean (Fig. 2b). Again, HRs for IG from MAD, MIMS, and Counts all followed a broadly similar pattern, with overlapping CIs. However, the dose response association was most linear for IG from MIMS with no plateau evident, while the HR for IG from Counts exhibited a local maximum around the mean, and HRs for IG from both Counts and MAD were flat above 1 SD. As was the case for the AvAcc, observed HRs for IG show greater consistency for UK Biobank than for Whitehall II.

Comparing AvAcc to IG, HRs tended to be higher for AvAcc below the mean than for IG below the mean, with HRs for IG decreasing more linearly throughout the range.

### Combined associations of high versus low PA volume and intensity with mortality risk

#### Whitehall II

**Fig. 3:**
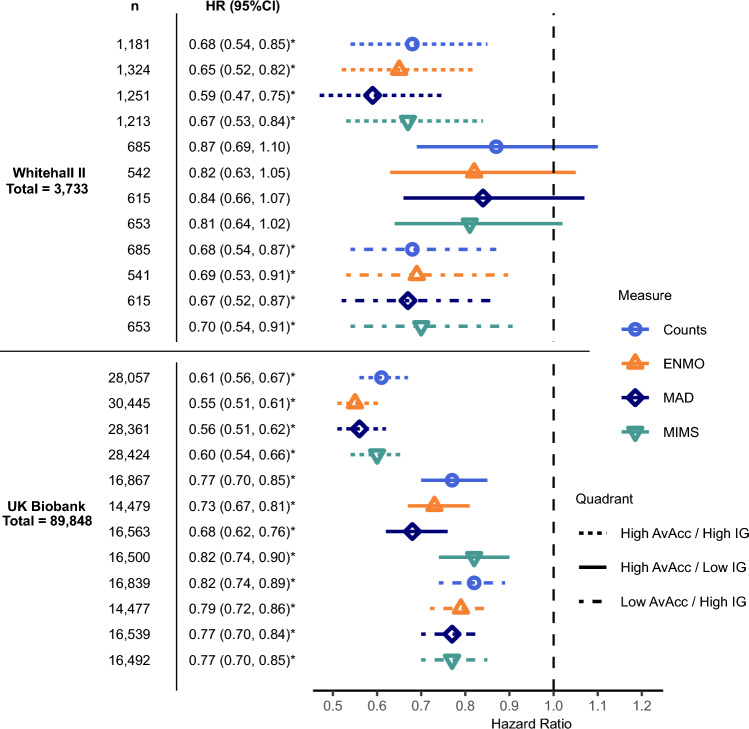
Hazard ratios for mortality according to quadrants of high/low AvAcc and IG. Count of participant per quadrant and hazard ratios with 95% confidence intervals (95%CI). Low and high correspond to lower and higher values than the median, respectively. Low AvAcc and low IG as reference. *Significantly different from reference at p < 0.05. AvAcc: Average Acceleration, IG: Intensity Gradient, ENMO: Euclidean Norm Minus One, MAD: Mean Amplitude Deviation, MIMS: Monitor Independent Movement Summary, High AvAcc/High IG: high Average Acceleration and high Intensity Gradient, High AvAcc/Low IG: High Average Acceleration and Low Intensity Gradient, Low AvAcc/High IG: Low Average Acceleration and High Intensity Gradient. Models adjusted for sex, season, ethnicity, employment status, smoking, alcohol consumption frequency, and sleep duration. See Supplementary Table S1 for details.

With the combination of low AvAcc and low IG as reference, the lowest hazard ratio with ENMO as the measure was for the combination of high AvAcc and high IG (0.65 (0.52, 0.82), Fig. 3). When AvAcc was low, but IG high, risk was also significantly lower (0.69 (0.53, 0.91)). Conversely, there was no significantly lower risk when AvAcc was high, but IG low (0.82 (0.63, 1.05)). The pattern of results was similar for MAD, MIMS, and Counts.

#### UK Biobank

The lowest risk with ENMO as the measure was again for the combination of high AvAcc and high IG (0.55 (0.51, 0.61)). In contrast to Whitehall II, mortality risk was lower if either AvAcc or IG was high, with the risk difference tending to be greater when AvAcc was high but IG low (0.73 (0.67, 0.81)) than vice versa (0.79 (0.72, 0.86)). The pattern of results was similar to ENMO for MAD and Counts, with risk differences tending to be larger or similar for MAD, but smaller or similar for Counts. For MIMS, the risk was lower for low AvAcc with high IG (0.77 (0.70, 0.85)) than vice versa (0.82 (0.74, 0.90)) akin to the results in Whitehall II.

For both continuous and categorical models, sensitivity analyses including hypothesised mediators as model covariates tended to attenuate the associations but otherwise displayed a similar pattern of results. Excluding participants aged under 60 in UK Biobank did not materially alter results (n cases/total = 56,767/89,848; median (IQR) age at death = 74.5 (70.9, 77.9)). Results are included in Supplementary Figures S4–S8.

## Discussion

Risk of all-cause mortality is lower for higher values of either of AvAcc and IG and appears largely similar when AvAcc and IG are derived from ENMO^2,5^ or when derived from MAD, MIMS, or Counts, with IG adjusted for each of the latter measures^23^. These findings suggest that, for sufficiently large samples, similar associations between PA and mortality would be obtained, irrespective of whether ENMO, MAD, MIMS or Counts are used as the underlying measure. However, while a difference in the relative impact of AvAcc and IG was evident between cohorts for ENMO, MAD, and Counts, this was not evident for MIMS. Overall, results from the combination of ENMO and MAD, both derived from simpler mathematical formulations, tended to be more consistently similar than from combinations including either of Counts or MIMS, both derived from more complex, layered formulations.

### Consistency of combined associations of AvAcc and IG with all-cause mortality across ENMO, MAD, MIMS, and Counts

Irrespective of measure, cohort, or AvAcc, IG was associated with reduced risk of mortality. This indicates that for a given volume of PA, accumulating that volume through higher intensities of PA appear to confer an added benefit. In this aspect, our results are consistent with a 2023 study in Whitehall II indicating that either of AvAcc and IG, derived from ENMO, add predictive value for mortality risk when added to a model including the other^27^, and to multiple UK Biobank studies investigating associations of AvAcc and IG to mortality risk or incident CVD^2,3^, or other volume and intensity measures to mortality risk or incident CVD^3,4^, all using a filtered variant of ENMO, standard in UK Biobank^13^. A recent analysis in NHANES found similar results, again using ENMO as the measure^5^. Willems et al. found mixed results for associations of IG with cardiometabolic variables between ENMO, MAD, and Counts using the original bins unadjusted for all measures^18^. The greater consistency between measures in the present study may partly be ascribed to population, larger sample sizes, and choice of outcome, but also likely indicates improved comparability conferred by using measure-specific adjustments when deriving IG. That consistency may increase with sample size is supported by the fact that results were more consistent in the UK Biobank than in Whitehall II.

That AvAcc appears to be more strongly associated with all-cause mortality in UK Biobank than IG is consistent with the one similar analysis^2^. In contrast, in Whitehall II, IG was the primary driver for lower mortality risk. In this aspect, our results differ with the Whitehall II analysis mentioned in the prior paragraph, which showed a stronger association for AvAcc than for IG^27^. Those results were based on data with shorter follow-up period, and consequently, a lower proportion of cases (10.3% vs. 15.1% in the present study). In addition, models included either AvAcc or IG separately and were not mutually adjusted, meaning the findings cannot be directly compared.

The cause for this discrepancy between the Whitehall II and UK Biobank cohorts is unclear. Device differences, including brand and the relatively similar sampling frequencies of 85.7, and 100 Hz for Whitehall II, and UK Biobank, respectively, are unlikely to be a primary cause^36^, especially in the context of 24-h measures like AvAcc and IG. AvAcc was previously found to be about 10 percent higher at the dominant, compared to the non-dominant, wrist using the GENEActiv and Axivity devices, while IGs were found to be equivalent, with high ICCs for both^37^, and high shared variances at the epoch level in a separate study^38^. A slightly higher AvAcc, relative to IG, within UK Biobank, compared to Whitehall II, could potentially be part of the of the reason for the discrepancy. Though, given the high ICCs reported between wrists^37^ and high shared variance^38^, the effect is likely to be small. The Whitehall II cohort was older with a correspondingly higher age at death. In an older cohort, higher IG may indicate better general health and functional capacity, through the ability to accumulate PA volume at higher intensities of PA, which would increase the impact of higher IG by proxy. The percentage of respiratory and CVD-related deaths were similar between the Whitehall II and UK Biobank cohorts, though percentage of cancer-related deaths were higher in UK Biobank. Excluding UK Biobank participants under 60 years old at time of accelerometer measurement did not meaningfully change the pattern of results, however, age at death (74.5 (70.9, 77.9)) and percentage of cancer-related deaths (54.3%) were similar to the full cohort. The measured PA levels of the cohorts reflect the age difference, with UK Biobank more active on average than Whitehall II. Considering Schwendinger et al.^5^, it is unlikely, however, that higher activity levels or age at death could explain the discrepancy, as IG had the strongest association with all-cause mortality in NHANES (2011–2014), as was the case in the present study for Whitehall II, despite the NHANES cohort being more active than either the Whitehall II or UK Biobank cohorts, and showing an age at death similar to UK Biobank^5^. Schwendinger et al. did not report proportion of cancer-related deaths in NHANES.

### Relative agreement of AvAcc and IG derived from ENMO, MAD, MIMS and Counts

Our analyses show the strongest correlations between ENMO and MAD measures for both AvAcc and IG in both cohorts, but slightly weaker correlations for remaining combinations varying between cohorts. The correlations between ENMO and MAD and between MIMS and Counts are very similar between cohorts. This may reflect that ENMO and MAD are both based on the epoch mean of the vector magnitude of acceleration, while MIMS and Counts are in arbitrary units derived from more complex algorithms. The pattern of results, i.e. ENMO and MAD strongly correlated to each other, and less correlated to either of MIMS or Counts, with remaining combinations varying somewhat between datasets, is consistent with other studies^18,20,23,38^, and the strong correlation between ENMO and MAD, in particular, was expected based on their similarities^19^.

### Strengths and limitations

Notable strengths of this study are the inclusion of both Whitehall II and UK Biobank data, providing important context to the generalisability of results across common differences found between similar cohorts, relating to both cohort characteristics and measurement protocol. UK Biobank in particular is a very large dataset in which multiple analyses examining associations of PA volume and intensity distribution with mortality and other health outcomes provide a rich source of analyses to compare against. Notable limitations include both cohorts being from the UK and being predominantly White, older, relatively healthy, and less socioeconomically deprived^25,39^. As a result, generalisability of results to diverse geographic, ethnic, and cultural contexts is limited, although this likely impacts PA-mortality associations per se more so than the comparability between measures. Only either wrist was included as wear site, and only one epoch length (5 s) was tested, making generalisability to other wear sites and epoch lengths limited. It is worth noting though, that while epoch length does impact IG, it does not impact AvAcc when based on the measures included herein. Considering Skinner et al.^33^, shorter epoch durations would likely lead to slightly lower p-values for IG-health associations and potentially slightly stronger effects, while the opposite is true for longer durations. As the present analysis had sufficient power to detect IG-health associations for both cohorts with 5 s epochs, shorter epochs would not be expected to change the results significantly. Longer duration epochs would be expected to make the IG progressively less sensitive. Correlations between AvAcc and IG were also reported to increase with longer epoch durations. Given the mathematical derivation of the IG, this result is expected, meaning that the IG necessitates a relatively short epoch to be valid, and to differentiate meaningfully from AvAcc. This is especially relevant if included in mutually adjusted models with AvAcc as in the present analysis. Differences between cohorts are likely impacted by differences between wrists, device, and sampling rate, though the magnitudes of any such impacts are expected to be small. Of these, the difference between wrists is likely the biggest limitation. Assuming the absolute magnitude of additional non-PA wrist activity reflected in measurement from the dominant wrist is mostly independent of PA levels, it is likely to make up a larger part of measured accelerations in participants with low PA. This could mask differences in PA, potentially leading to some degree of relative underestimation of PA-mortality associations at the lower end of the PA spectrum, when assessed at the dominant wrist, likely more so for AvAcc than for IG. As associations were higher in UK Biobank than Whitehall II, and given the high ICCs between wrists reported by Rowlands et al.^37^, the effect is likely to be small in general. AvAcc from both ENMO and MAD failed tests of proportionality in the continuous models in the UK Biobank cohort. Schoenfeld residuals showed that the time interaction was negligible for both, and patterns of results remained consistent with the main analysis when including a (slightly positive) time-dependent coefficient for AvAcc (Supplementary Figure S9). Due to the time-dependent coefficient being small (HR: 1.01 (1.01, 1.02) per year, centred on mean age at follow-up or death of 70.3 years for both ENMO and MAD) and proportionality of hazard for AvAcc being met in the remaining six models (all four in Whitehall II, MIMS and Counts in UK Biobank), main results were based on models without the time-dependent coefficient to keep all compared models consistent. It is worth noting, however, that HRs for AvAcc derived from either ENMO or MAD in UK Biobank should be interpreted as averages over time. Some bias due to differences in covariate measurement between the two cohorts, and possible other covariates that were not included, cannot be ruled out. The dichotomisation of high versus low AvAcc or IG is data-driven, meaning that a lack of representativeness in terms of AvAcc and IG would skew the split relative to the underlying population. Further, it discards the resolution provided by continuous data. This limitation is mitigated by presentation and interpretation of models based on the full continuous data alongside the dichotomised models. Using ENMO-based thresholds alone for data trimming leads to potential for some outliers remaining in other measures. With only 15 participants excluded in UK Biobank and none in Whitehall II due to unrealistic ENMO values (> 100 m*g*), any impact from using ENMO-based thresholds for data trimming would likely be minimal. For the < 0.5th percentile and > 99.5th percentile exclusions there could be more of an impact, however alternative methods each present their own limitations. Applying exclusions for each measure individually would lead to discrete data sets for each analysis, while applying exclusions based on all measures on the same data set would lead to more exclusions. Which option is most valid is debatable. Finally, it is worth noting, that the present analysis was not designed primarily to make inferences about the relative impacts of volume and intensity distribution on mortality risk, but to assess consistency of mortality-risk associations between measures. The ecological validity of such inferences, particular in terms of PA guidelines, exclusively based on mutually adjusted survival models, is limited due to the inherent interconnected nature of volume and intensity distribution of PA, meaning that any recommended added PA will almost inevitably result in changes in both. Comparisons to other studies included here are meant to give context to the applicability of our results, and to serve as an indicator of the face validity of the survival analyses on which they are based. Future studies should further explore the similarities and differences between common epoch summary measures and between other common differences in accelerometer data processing.

## Conclusions

Our results indicate that trends of associations of AvAcc and IG with all-cause mortality are likely to be similar between studies utilising ENMO, MAD, MIMS, or Counts as their measure. However, it is important that IG is calculated appropriately for the measure selected by using the proposed adjustments. Our results will enable researchers to have confidence in comparing conclusions from studies that utilise different measures when investigating associations of AvAcc and IG with health. This is highly relevant as all these measures are deployed in large-scale national studies. The main caveats to this are, first, that numerical estimates, and whether results reach statistical significance at a given sample size, may differ somewhat, and second, that results from ENMO and MAD tend to be more consistently similar than from combinations including either of Counts or MIMS. In addition, lack of agreement and comparability in device-based PA measurement methodology has meant that public health guidelines to date have remained reliant primarily on self-report measures^40^. With all-cause mortality as a critical outcome for PA guideline development^41^, our results should strengthen the confidence of not only researchers, but also public health officials and policy makers in incorporating device-based measurement, which can enable more accurate and detailed understanding of PA, and in turn more accurate and detailed PA surveillance efforts and PA guidelines.

## Supplementary Information

Supplementary Information.

## Data Availability

Data, protocols, and other metadata of the Whitehall II study are available to the scientific community via the Whitehall II study data sharing portal (https://www.ucl.ac.uk/psychiatry/research/mental-health-older-people/whitehall-ii/data-sharing). The database supporting the conclusions of this article is available from UK Biobank project site, subject to registration and application process. Further details can be found at https://www.ukbiobank.ac.uk. Statistical analysis code is available on GitHub at https://github.com/henrikeckmann.
