# Supplementary material for: Impact of accelerometer epoch summary measure on associations between physical activity and all-cause mortality in Whitehall II and UK Biobank
Source: Sci Rep. 2025 Dec 6;16:761. doi: 10.1038/s41598-025-30237-5 (PMC12780095; doi:10.1038/s41598-025-30237-5)

**Supplementary Figure S1. Bayesian information criterion for all continuous models using natural splines with three through seven internal knots.** BIC: Bayesian Information Criterion, UKBB: UK Biobank, ENMO: Euclidean Norm Minus One, MAD: Mean Amplitude Deviation, MIMS: Monitor Independent Movement Summary


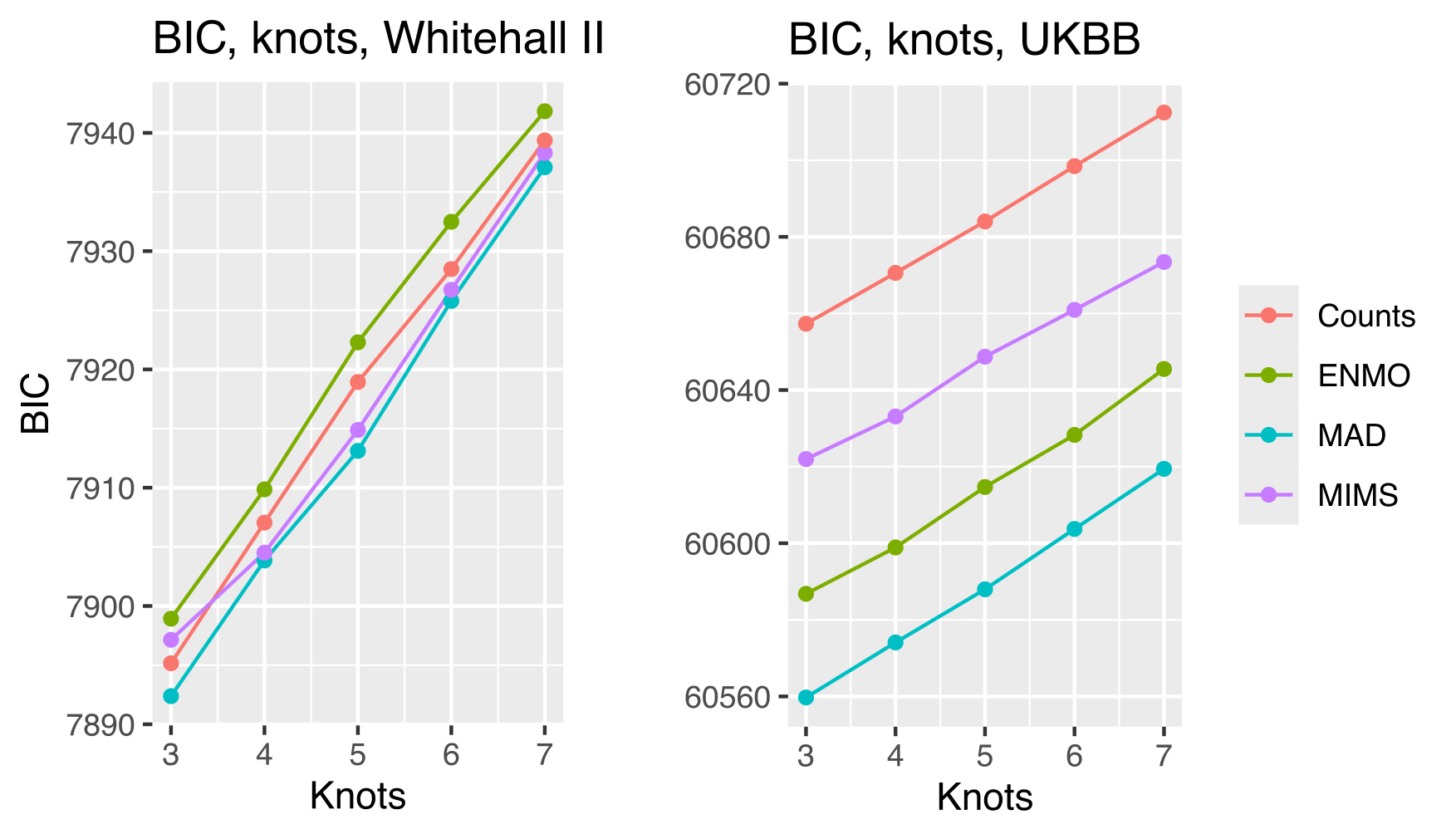


**Supplementary Figure S2. Directed Acyclic Graph showing hypothesized associations between model covariates.** CVD: Cardiovascular disease. Potential mediators and confounders: covariates hypothesised to be either confounders or mediators. See supplementary Table S1 for model and covariate details.


**Supplementary Section S1. Epoch Summary Measures**

**Counts**

The Counts algorithm published by Neishabouri et al. in 2022(Neishabouri et al., 2022) is essentially a digital emulation of the analog signal chain of the CSA 7164 from 1996(Tryon & Williams, 1996). Because of this it is more complex than the algorithms for ENMO or MAD (discussed below), and not all the mathematical notation is included here. The algorithm consists of the following steps, for each axis(Neishabouri et al., 2022):

- Assert that sampling frequency is between 30 and 100 Hz and a multiple of 10 Hz
- If sampling frequency is not a multiple of 30, up-sample to a multiple of 30 and low pass filter.
- Down-sample to 30 Hz by keeping every $n$th sample, where $n$ is the up-sampled frequency divided by 30.
- Band-pass filter with maximum gain at 0.749 Hz, with a slope reaching -6 dB at 0.212 Hz and 2.148 Hz.
- Rescale filtered signal to replicate range of the CSA 7164.
- Rectify signal.
- Threshold signal above 128 and below 4 (any value above 128 is set to 128, any below 4 is set to 0) round down to integer values.
- Down-sample to 10 Hz, low pass filter, and round down to integer values.
- Sum over given epoch.

This produces uniaxial Counts for each axis. For triaxial Counts one last step is added, taking the vector magnitude (the Euclidean norm, $\left\| (v_{1},v_{2},v_{3}) \right\|_{2}=\sqrt{v_{1}^{2}+v_{2}^{2}+v_{3}^{2}}$) of the Counts from the individual axes per epoch.

**Euclidean Norm Minus One**

The Euclidean Norm Minus One was the first of the widely used open-source epoch summary measures, developed by van Hees et al.(van Hees et al., 2013) in 2013, and remains one of the simplest. ENMO is the default measure in GGIR. With $A_{i}=\left( x_{i},y_{i},z_{i} \right)$ as the $i$th sample of the triaxial raw signal vector timeseries, $j$ as the index of the first sample in a given epoch, and $N$ as the number of samples per epoch, ENMO is the epoch mean of the Euclidean norm minus one, hence the name, with negative values truncated to zero(van Hees et al., 2013).

$$1. ENMO=\frac{1}{N}\sum_{i=j}^{j+N-1} \max\left( \left\| A_{i} \right\|_{2}-1, 0 \right)=\frac{1}{N}\sum_{i=j}^{j+N-1} \max\left( \sqrt{x_{i}^{2}+y_{i}^{2}+z_{i}^{2}}-1, 0 \right)$$

Since ENMO is based on the three-dimensional Euclidean norm, including assumptions about the magnitude of the gravitational component in three dimensions, ENMO exists only as a triaxial measure. Subtracting 1 *g* is meant to account for the static component of the signal due to gravity.

**Mean Amplitude Deviation**

Mean Amplitude Deviation was proposed as an epoch level measure in 2015 by Vaha-Ypya et al. based on an experimental comparison of a variety of candidate measures in terms of classifying movement intensities(Vähä-Ypyä et al., 2015). Like ENMO it is a mathematically simple measure based on the Euclidean norm of a triaxial accelerometry signal, and it does not employ filtering or truncation, but unlike ENMO, Neishabouri counts, or MIMS, it is a measure of variability rather than magnitude as it is simply the mean absolute deviation around the mean (also called average absolute deviation) applied to accelerometer epochs. Using the same notation as for ENMO, MAD is the mean distance of the Euclidean norm of each triaxial sample in an epoch to the epoch mean(Vähä-Ypyä et al., 2015).

$$2. MAD=\frac{1}{N}\sum_{i=j}^{j+N-1} \left( \left\| A_{i} \right\|_{2}-\frac{1}{N}\sum_{i=j}^{j+N-1} \left\| A_{i} \right\|_{2} \right)$$

MAD does not directly account for the gravitational component but instead assumes that the gravitational component is purely static, meaning that it will not be reflected in a measure of variability, and that any dynamic component is due to movement.

**Monitor Independent Movement Summary**

The Monitor Independent Movement Summary is, like Neishabouri counts, a more complex algorithm, and like Neishabouri counts can be either uniaxial or triaxial. Uniquely, MIMS tries to account for differences in accelerometer specifications by interpolating data with lower sampling frequencies to 100 Hz, and extrapolating data that may have been clipped by the device’s dynamic range. The algorithm for each axis is as follows(John et al., 2019).

- Linear interpolation to 100 Hz.
- Extrapolation of points near the ends of device dynamic range.
- Bandpass filter, 0.2-5 Hz.
- Integration.
- Truncation of values below ${10}^{-3}$ to zero.

Like for Neishabouri counts an extra step is added to go from uniaxial to triaxial MIMS, but instead of using the Euclidean norm MIMS sums the axes together (also referred to as the Manhattan norm). Note that the paper seems to imply that truncation happens after triaxial aggregation but in the source code of the MIMSunit R package(*CRAN: Package MIMSUnit*), developed by one of the authors of the paper proposing MIMS, the order is as described here. MIMS is not implemented into GGIR.

**References**

*CRAN: Package MIMSUnit*. Retrieved 18-08 from <https://CRAN.R-project.org/package=MIMSunit>

John, D., Tang, Q., Albinali, F., & Intille, S. (2019). An Open-Source Monitor-Independent Movement Summary for Accelerometer Data Processing. *J Meas Phys Behav*, *2*(4), 268-281. <https://doi.org/10.1123/jmpb.2018-0068>

Neishabouri, A., Nguyen, J., Samuelsson, J., Guthrie, T., Biggs, M., Wyatt, J., Cross, D., Karas, M., Migueles, J. H., Khan, S., & Guo, C. C. (2022). Quantification of acceleration as activity counts in ActiGraph wearable. *Sci Rep*, *12*(1), 11958. <https://doi.org/10.1038/s41598-022-16003-x>

Tryon, W. W., & Williams, R. (1996). Fully proportional actigraphy: A new instrument. *Behavior Research Methods, Instruments, & Computers*, *28*(3), 392-403. <https://doi.org/10.3758/BF03200519>

Vähä-Ypyä, H., Vasankari, T., Husu, P., Suni, J., & Sievänen, H. (2015). A universal, accurate intensity-based classification of different physical activities using raw data of accelerometer. *Clin Physiol Funct Imaging*, *35*(1), 64-70. <https://doi.org/10.1111/cpf.12127>

van Hees, V. T., Gorzelniak, L., Dean León, E. C., Eder, M., Pias, M., Taherian, S., Ekelund, U., Renström, F., Franks, P. W., Horsch, A., & Brage, S. (2013). Separating movement and gravity components in an acceleration signal and implications for the assessment of human daily physical activity. *PLoS One*, *8*(4), e61691. <https://doi.org/10.1371/journal.pone.0061691>

**Supplementary Table S1. Model and covariate specifications

Model specifications**

|  | **Whitehall II** |  | **UK Biobank** | | **Variable type** |
| --- | --- | --- | --- | --- | --- |
| **Exposures** | |  |  | |  |
|  | AvAcc, spline 3 internal knots |  | AvAcc, spline 3 internal knots | | continuous |
|  | IG, spline 3 internal knots |  | IG, spline 3 internal knots | | continuous |
|  | AvAcc/IG high/low split |  | AvAcc/IG high/low split | | categorical |
| **Base model covariates** | |  |  | |  |
|  | Sex |  | Sex | | categorical |
|  | Season spring, spline 1 internal knot |  | Season spring, spline 1 internal knot | | continuous |
|  | Season winter, spline 1 internal knot |  | Season winter, spline 1 internal knot | | continuous |
|  | Ethnicity |  | Ethnicity | | categorical |
|  | Employment status |  | Employment status* | | categorical |
|  | Smoking status |  | Smoking status* | | categorical |
|  | Alcohol frequency |  | Alcohol frequency | | categorical |
|  | Self-reported sleep duration |  | Self-reported sleep duration | | categorical |
| **Additional covariates for sensitivity analysis** | | | |  |  |
|  | No. of medications |  | No. of medicines | | continuous |
|  | BMI classification |  | BMI classification | | categorical |
|  | Prevalent CVD |  | Prevalent CVD | | categorical |
|  | Other diseases |  | Other diseases* | | continuous |
|  | Prevalent cancer* |  | Prevalent cancer* | | categorical |
|  |  |  | Long-standing illness, disability or infirmity* | | categorical |

**Covariate specifications**

|  | Season spring | | $sin(2\pi\cdot day of year/365.25)$ | | | | |
| --- | --- | --- | --- | --- | --- | --- | --- |
|  | Season winter | | $cos(2\pi\cdot day of year/365.25)$ | | | | |
| **Whitehall II** | day of year: first day of month of accelerometer measurement | | | | | | |
| **UK Biobank** | day of year: first day of accelerometer measurement | | | | | | |
|  |  |  | |  | |  | |
|  | **Prevalent CVD** | | | | **HES** | | **Screening** |
| **Whitehall II** | Composite of | | | |  | |  |
|  |  | CHD | | ICD-10: I20-25, OCPS-4: K40-50, K75, U19 | | ECGs, visit 1-11 | |
|  |  | Heart failure | | ICD-10: I50 | |  | |
|  |  | Stroke | | ICD-10: I60-64 | | Questionnaire, visit 1-9 | |
|  |  |  | |  | |  | |
| **UK Biobank** |  |  | | ICD-10: I00-99 | | Baseline or most recent questionnaire | |
|  |  |  | |  | |  | |
|  | **Prevalent cancer** | | | | **HES** | | **Screening** |
| **Whitehall II** |  |  | | ICD-10: C97, except C44 | | | |
|  |  |  | |  | |  | |
| **UK Biobank** |  |  | | ICD-10: C00-97 | | Baseline or most recent questionnaire | |
|  |  |  | |  | |  | |
|  | **Other diseases** | | | | **HES** | | **Screening** |
| **Whitehall II** | Number of diagnoses among | | | |  | |  |
|  |  | Parkinson's | | ICD-10: G20 | |  | |
|  |  | COPD | | ICD-10: J41-44 | | | |
|  |  | Depression | | ICD-10: F32, F33 | | Use of antidepressant drugs, visit 1-11 | |
|  |  | Other mental disorders | | ICD-10: F06-09,  F20-31, F34-48,  F60-64, F67-69 | | | |
|  |  | Liver disease | | ICD-10: K70-79 | | | |
|  |  | Arthritis | | ICD-10: M05-06,  M15-19 | | | |
|  |  | Hypertension | | ICD-10: I10-15 | | Blood pressure measures, use of antihypertensive drugs, visit 1-11 | |
|  |  | T2D | | ICD-10: E11 | | Report by doctor, use of diabetic drugs, fasting glucose >= 7mmol/L, long-standing illness questionnaire, visit 1-11 | |
|  |  | Dementia | | ICD-10: F00-F03, F05.1, G30, and G31 | |  | |
|  |  |  | |  | |  | |
| **UK Biobank** | Number of diagnoses among | | | | | |  |
|  |  | CKD | |  | | Baseline or most recent questionnaire | |
|  |  | Diabetes | |  | | Baseline or most recent questionnaire, baseline clinical screening type 2 diabetes indication. Questionnaire does not distinguish between type. | |
|  |  | Hypertension | | | | Baseline or most recent questionnaire | |

* Covariate used to stratify baseline hazards
AvAcc: Average Acceleration, IG: Intensity Gradient, BMI: Body Mass Index, CVD: Cardiovascular disease, CHD: Coronary Heart Disease, ICD-10: International Statistical Classification of Diseases and Related Health Problems 10^th^ Revision, ECG: Echocardiogram, COPD: Chronic Obstructive Pulmonary Disease, T2D: Type 2 Diabetes, CKD: Chronic Kidney Disease

**Supplementary Figure S3. Exclusions flowchart.** Insufficient valid days: < 3 days of valid accelerometer wear, outliers: below/above the 0.5^th^/99.5^th^ percentile of either AvAcc or IG from ENMO, unrealistic values: AvAcc from ENMO >100 m*g*.


**Supplementary Figure S4. Hazard ratios for mortality according to a) AvAcc and b) IG from ENMO, MAD, MIMS, and Counts, Whitehall II including all covariates.** X-axis: Number of standard deviations above or below the mean, y-axis: Hazard ratio for all-cause mortality, shaded areas correspond to 95% confidence intervals. AvAcc: Average acceleration, IG: Intensity Gradient, ENMO: Euclidean Norm Minus One, MAD: Mean Amplitude Deviation, MIMS: Monitor Independent Movement Summary. Models adjusted for sex, season, ethnicity, employment status, smoking, alcohol consumption frequency, sleep duration, number of prescribed medications, body mass index, cardiovascular disease, cancer, number of other diseases, and long-standing illness, disability or infirmity (UK Biobank only). See Supplementary Table S1 for details.
**
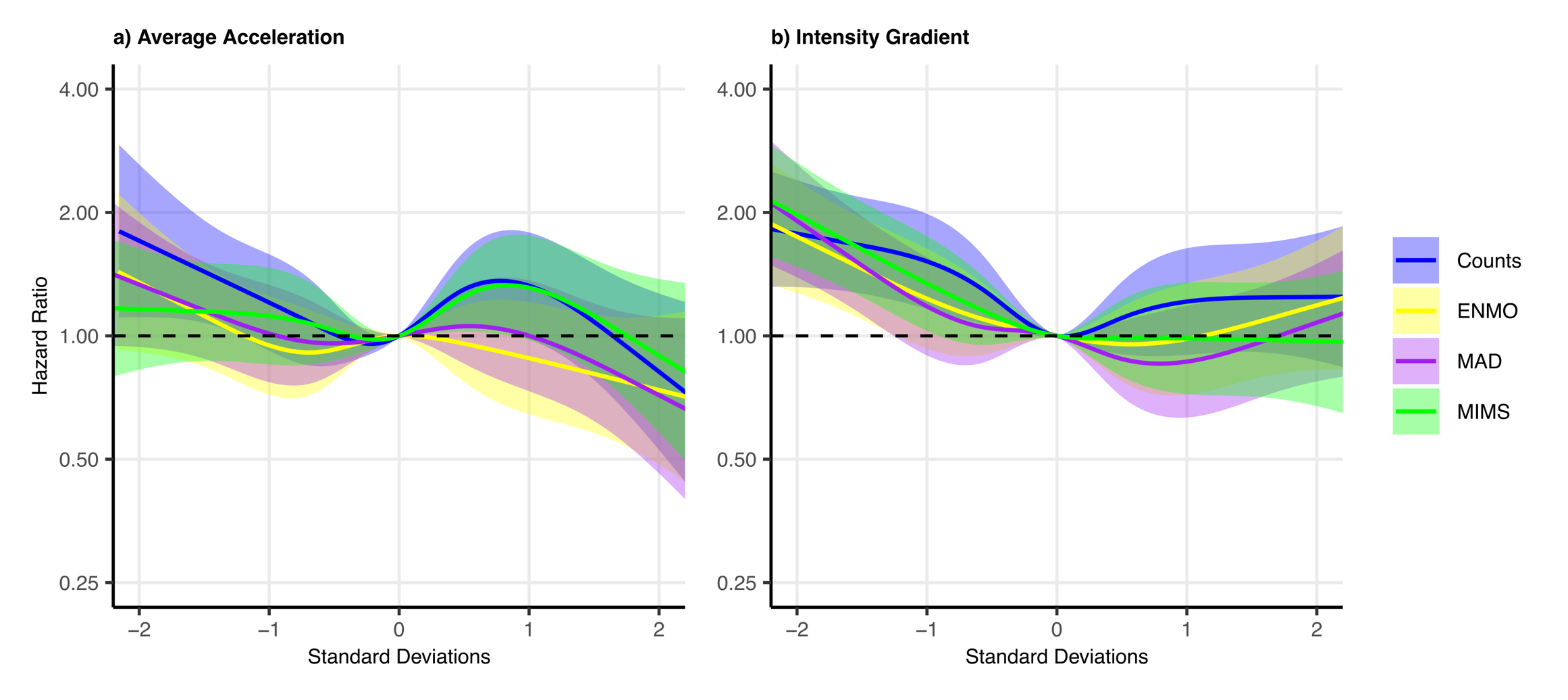
**

**Supplementary Figure S5. Hazard ratios for mortality according to a) AvAcc and b) IG from ENMO, MAD, MIMS, and Counts, UK Biobank including all covariates.** X-axis: Number of standard deviations above or below the mean, y-axis: Hazard ratio for all-cause mortality, shaded areas correspond to 95% confidence intervals. AvAcc: Average acceleration, IG: Intensity Gradient, ENMO: Euclidean Norm Minus One, MAD: Mean Amplitude Deviation, MIMS: Monitor Independent Movement Summary. Models adjusted for sex, season, ethnicity, employment status, smoking, alcohol consumption frequency, sleep duration, number of prescribed medications, body mass index, cardiovascular disease, cancer, number of other diseases, and long-standing illness, disability or infirmity (UK Biobank only). See Supplementary Table S1 for details.


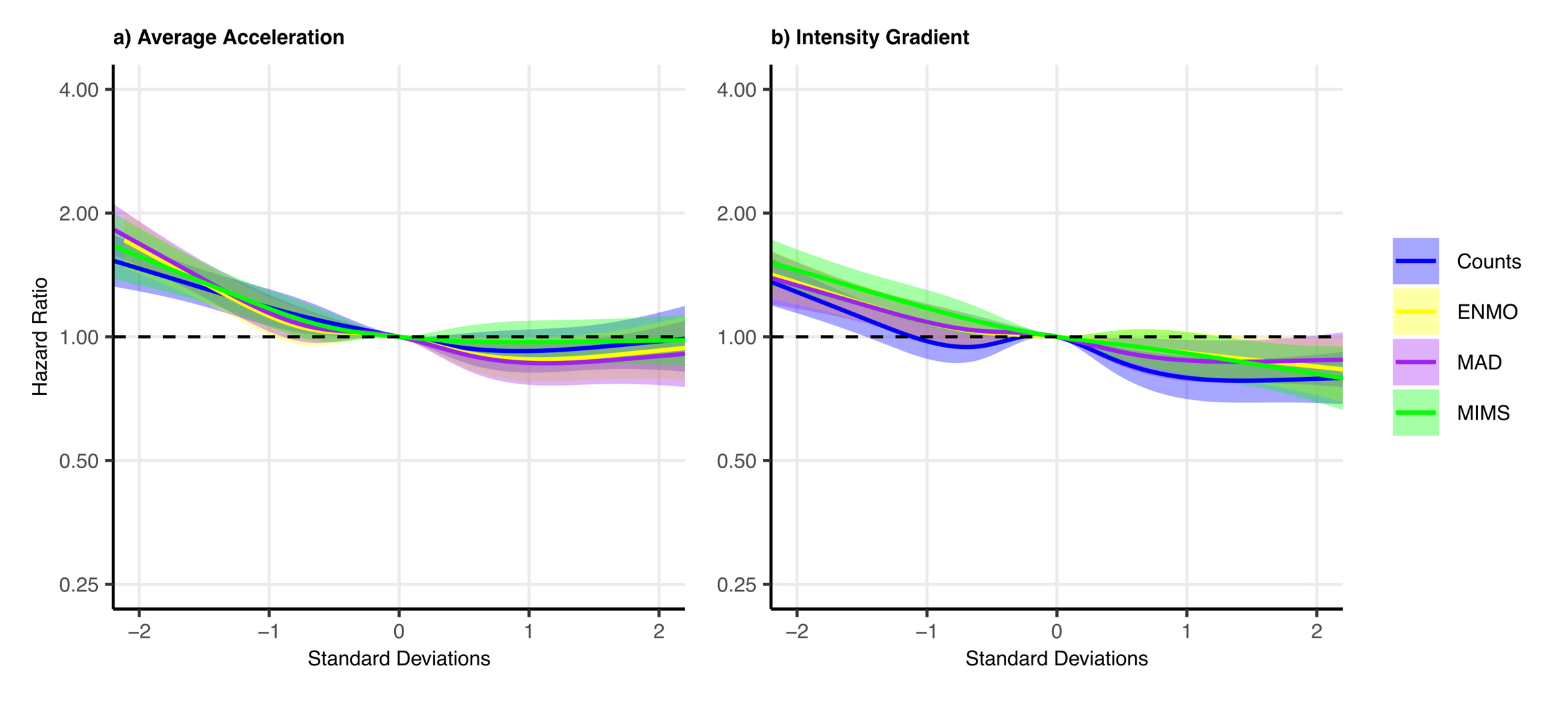


**Supplementary Figure S6. Hazard ratios for mortality according to a) AvAcc and b) IG from ENMO, MAD, MIMS, and Counts, UK Biobank, age >60 years.** X-axis: Number of standard deviations above or below the mean, y-axis: Hazard ratio for all-cause mortality, shaded areas correspond to 95% confidence intervals. AvAcc: Average acceleration, IG: Intensity Gradient, ENMO: Euclidean Norm Minus One, MAD: Mean Amplitude Deviation, MIMS: Monitor Independent Movement Summary. Models adjusted for sex, season, ethnicity, employment status, smoking, alcohol consumption frequency, and sleep duration. See Supplementary Table S1 for details.
**
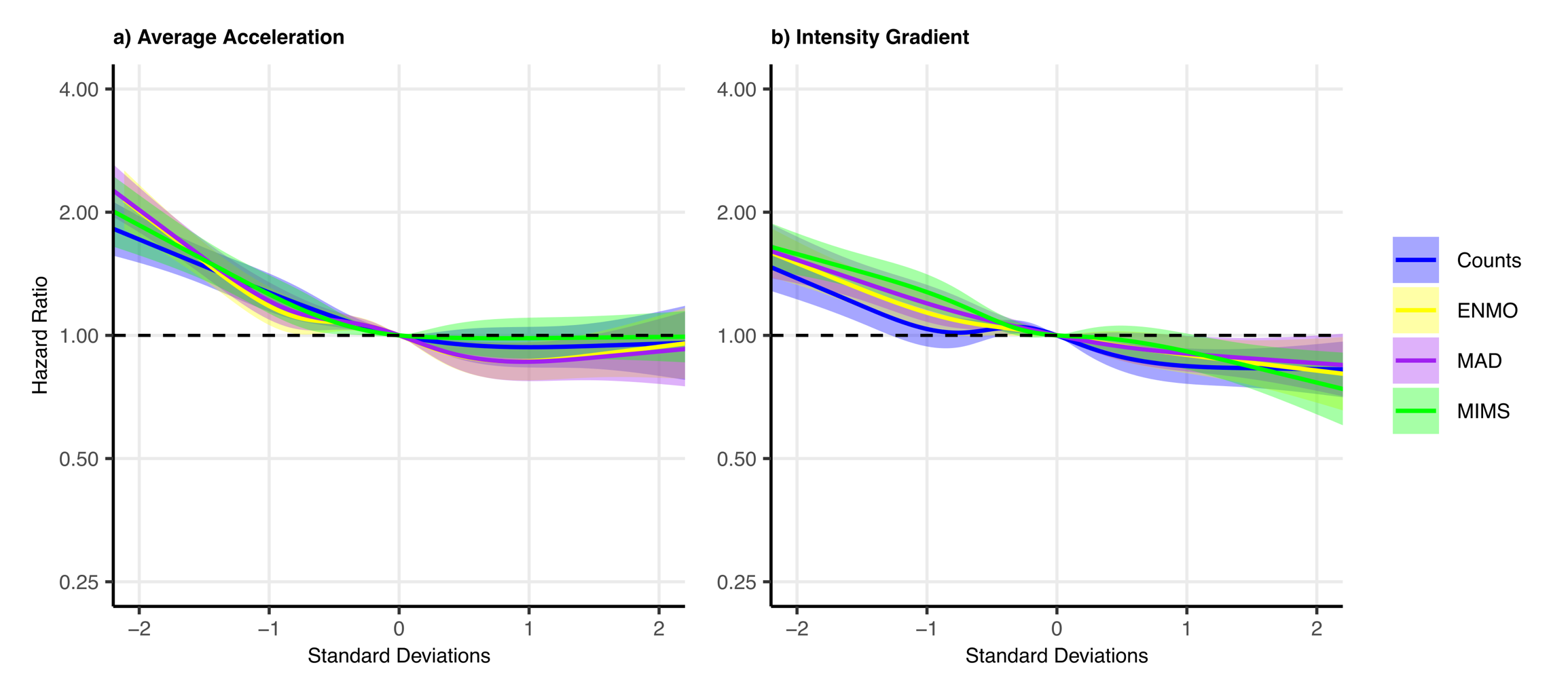
**

**Supplementary Figure S7. Hazard ratios for mortality according to quadrants of high/low AvAcc and IG, including all covariates.** Count of participant per quadrant and hazard ratios with 95% confidence intervals (95%CI). Low and high correspond to lower and higher values than the median, respectively. Low AvAcc and low IG as reference.
* Significantly different from reference at p<0.05.
AvAcc: Average Acceleration, IG: Intensity Gradient, ENMO: Euclidean Norm Minus One, MAD: Mean Amplitude Deviation, MIMS: Monitor Independent Movement Summary, High AvAcc / High IG: high Average Acceleration and high Intensity Gradient, High AvAcc / Low IG: High Average Acceleration and Low Intensity Gradient, Low AvAcc / High IG: Low Average Acceleration and High Intensity Gradient. Models adjusted for sex, season, ethnicity, employment status, smoking, alcohol consumption frequency, sleep duration, number of prescribed medications, body mass index, cardiovascular disease, cancer, number of other diseases, and long-standing illness, disability or infirmity (UK Biobank only). See Supplementary Table S1 for details.


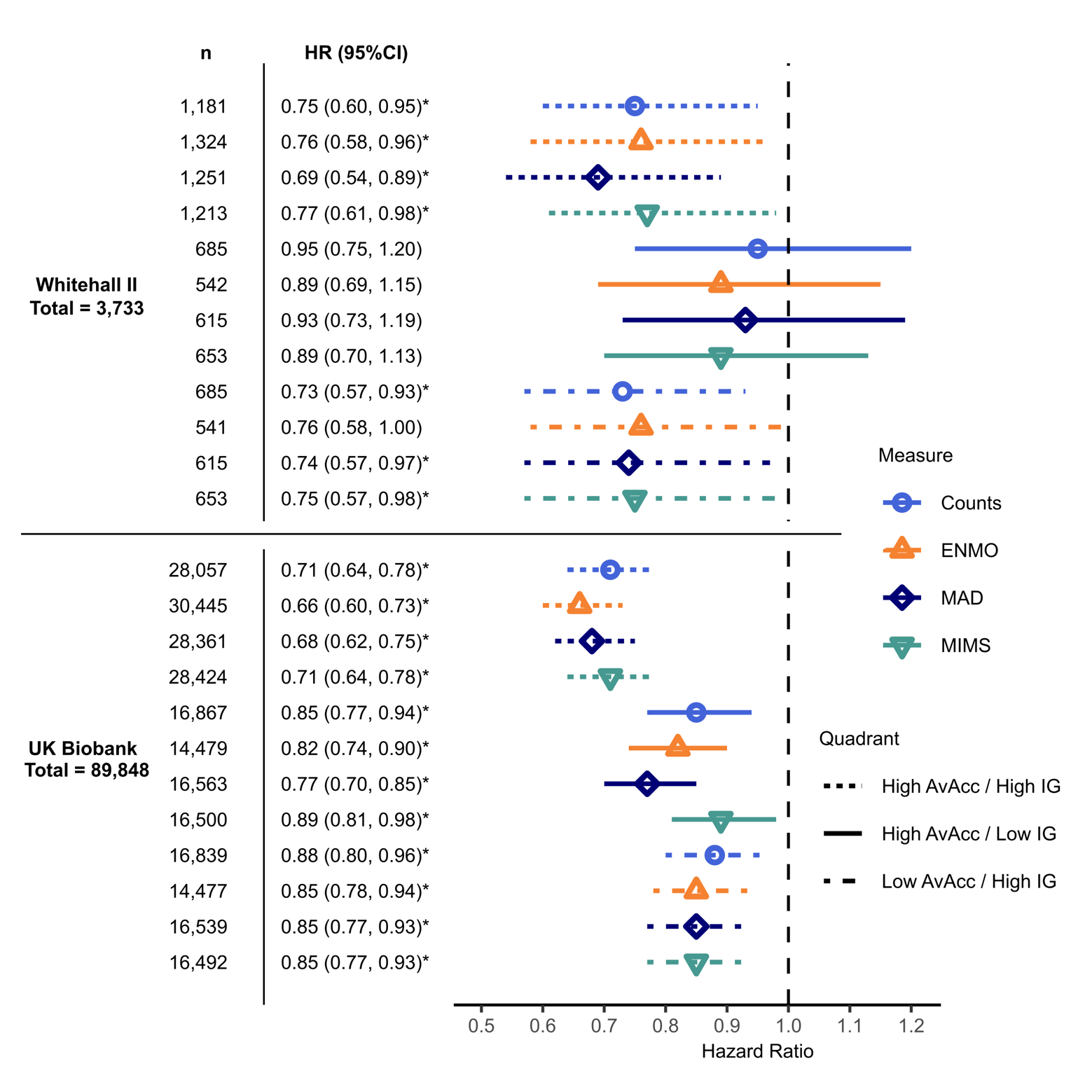


**Supplementary Figure S8. Hazard ratios for mortality according to quadrants of high/low AvAcc and IG, UK Biobank, age >60 years.** Count of participant per quadrant and hazard ratios with 95% confidence intervals (95%CI). Low and high correspond to lower and higher values than the median, respectively. Low AvAcc and low IG as reference.
* Significantly different from reference at p<0.05.
AvAcc: Average Acceleration, IG: Intensity Gradient, ENMO: Euclidean Norm Minus One, MAD: Mean Amplitude Deviation, MIMS: Monitor Independent Movement Summary, High AvAcc / High IG: high Average Acceleration and high Intensity Gradient, High AvAcc / Low IG: High Average Acceleration and Low Intensity Gradient, Low AvAcc / High IG: Low Average Acceleration and High Intensity Gradient. Models adjusted for sex, season, ethnicity, employment status, smoking, alcohol consumption frequency, and sleep duration. See Supplementary Table S1 for details.

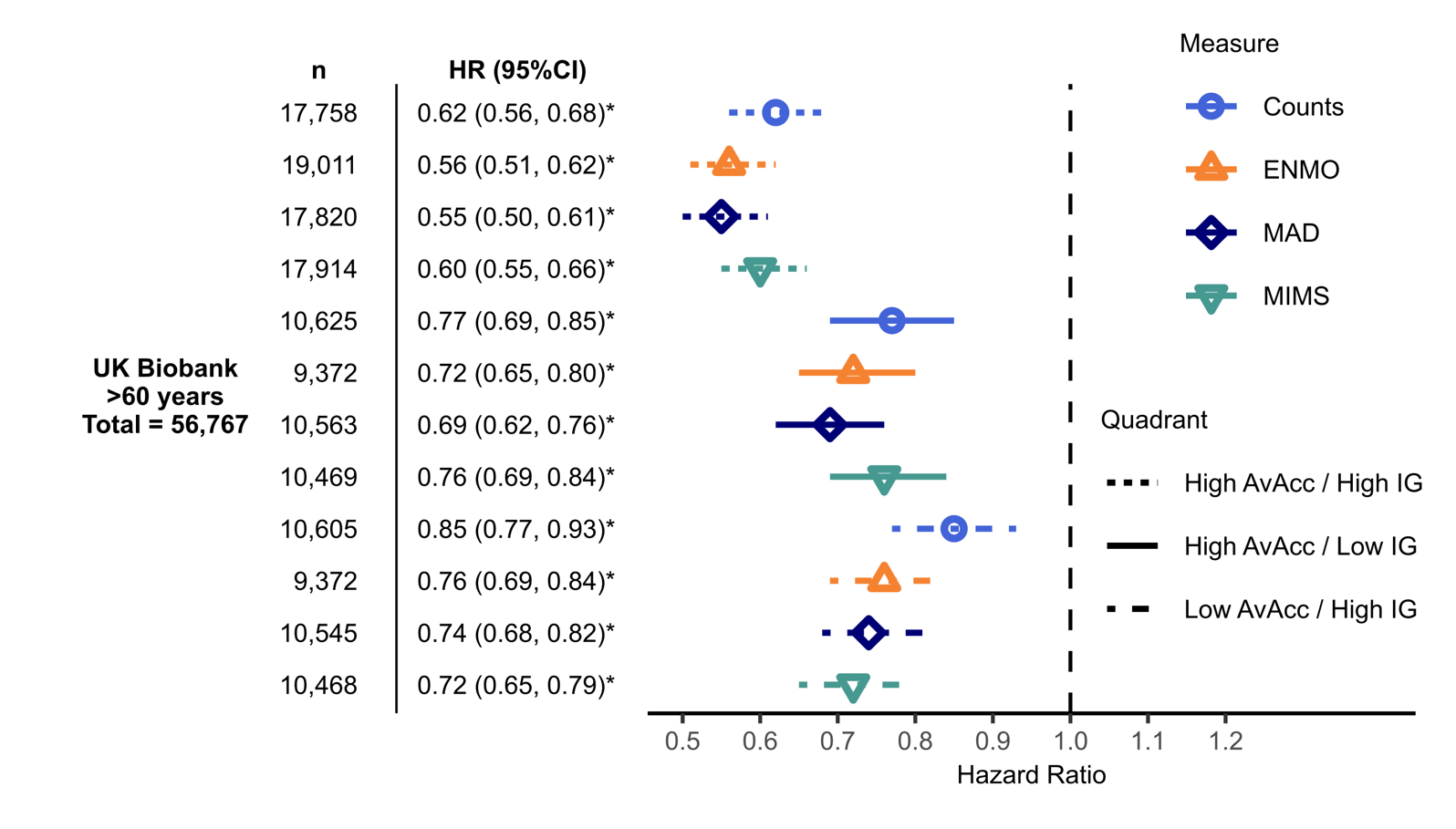


**Supplementary Figure S9. Hazard ratios for continuous models in UK Biobank for ENMO (top) and MAD (bottom) with (yellow) and without (blue) a time-dependent coefficient for AvAcc, a) Average Acceleration, b) Intensity Gradient.** X-axis: Number of standard deviations above or below the mean, y-axis: Hazard ratio for all-cause mortality, shaded areas correspond to 95% confidence intervals. ENMO: Euclidean Norm Minus One, MAD: Mean Amplitude Deviation.


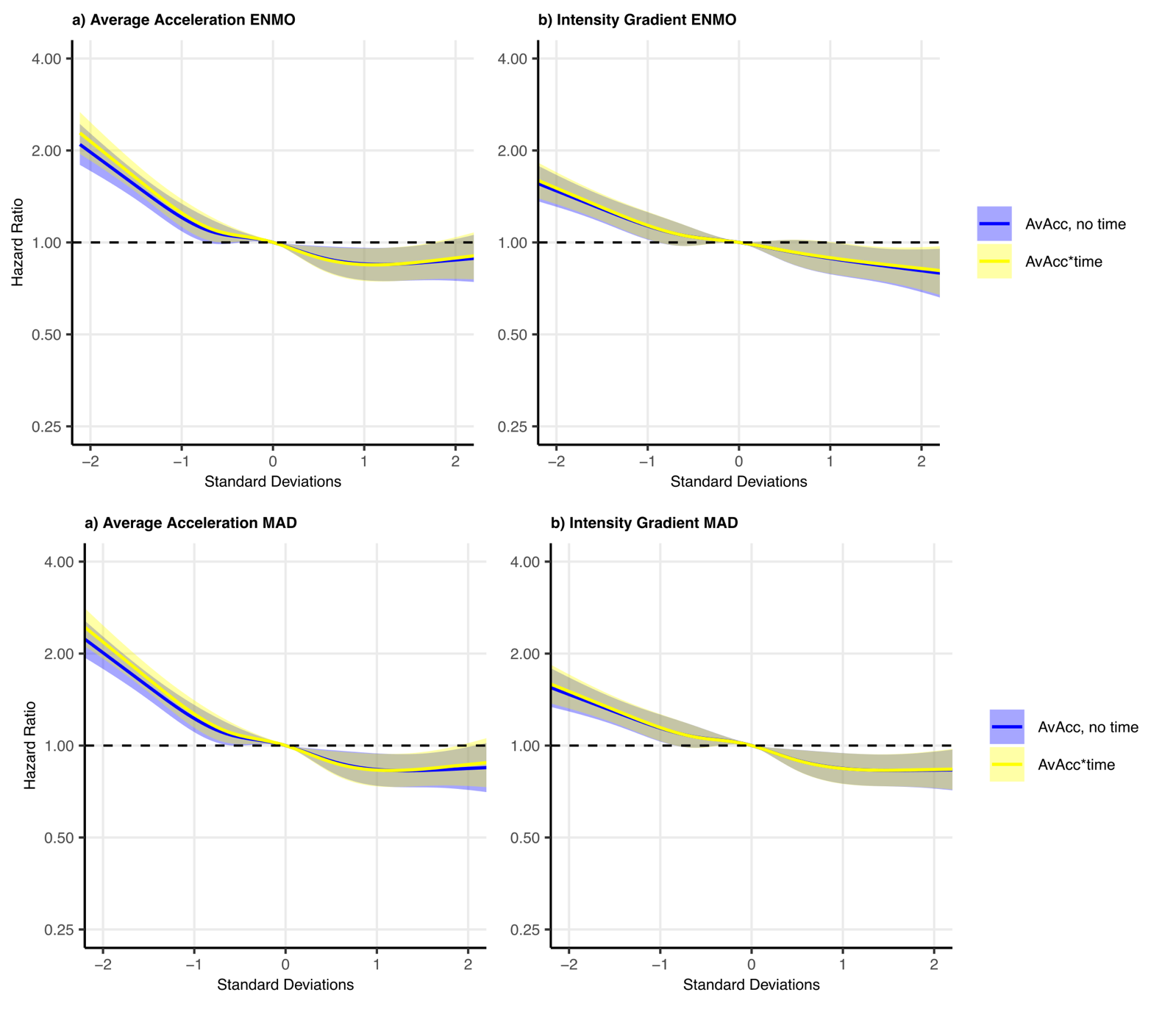

Supplement: Supplementary file 1 — Supplementary Information. [file 41598_2025_30237_MOESM1_ESM.docx]
